# ADAM17 Silencing in Mouse Colon Carcinoma Cells: The Effect on Tumoricidal Cytokines and Angiogenesis

**DOI:** 10.1371/journal.pone.0050791

**Published:** 2012-12-10

**Authors:** Sudipta Das, Maria Czarnek, Monika Bzowska, Renata Mężyk-Kopeć, Krystyna Stalińska, Barbara Wyroba, Jolanta Sroka, Jarosław Jucha, Dawid Deneka, Paulina Stokłosa, Justyna Ogonek, Melody A. Swartz, Zbigniew Madeja, Joanna Bereta

**Affiliations:** 1 Department of Cell Biochemistry, Faculty of Biochemistry, Biophysics and Biotechnology, Jagiellonian University, Kraków, Poland; 2 Department of Cell Biology, Faculty of Biochemistry, Biophysics and Biotechnology, Jagiellonian University, Kraków, Poland; 3 Institute of Bioengineering and Swiss Institute of Experimental Cancer Research (ISREC), Ecole Polytechnique Fédérale de Lausanne, Lausanne, Switzerland; Kaohsiung Chang Gung Memorial Hospital, Taiwan

## Abstract

ADAM17 (a disintegrin and metalloprotease 17) is a major sheddase for numerous growth factors, cytokines, receptors, and cell adhesion molecules and is often overexpressed in malignant cells. It is generally accepted that ADAM17 promotes tumor development via activating growth factors from the EGF family, thus facilitating autocrine stimulation of tumor cell proliferation and migration. Here we show, using MC38CEA murine colon carcinoma model, that ADAM17 also regulates tumor angiogenesis and cytokine profile. When ADAM17 was silenced in MC38CEA cells, *in vivo* tumor growth and *in vitro* cell motility were significantly diminished, but no effect was seen on *in vitro* cell proliferation. ADAM17-silencing was accompanied by decreased *in vitro* expression of vascular endothelial growth factor-A and matrix metalloprotease-9, which was consistent with the limited angiogenesis and slower growth seen in ADAM17-silenced tumors. Among the growth factors susceptible to shedding by ADAM17, neuregulin-1 was the only candidate to mediate the effects of ADAM17 on MC38CEA motility and tumor angiogenesis. Concentrations of TNF and IFNγ, cytokines that synergistically induced proapoptotic effects on MC38CEA cells, were significantly elevated in the lysates of ADAM17-silenced tumors compared to mock transfected controls, suggesting a possible role for ADAM17 in host immune suppression. These results introduce new, complex roles of ADAM17 in tumor progression, including its impact on the anti-tumor immune response.

## Introduction

Protein ectodomain shedding, the proteolytic release of extracellular domains of transmembrane proteins, is a process that modifies communication between cells as well as their interactions with extracellular environment and is thus crucial for various aspects of the cell biology. Proteases of ADAM family have emerged as major sheddases. Although there is a significant redundancy in the substrate recognition across the ADAM family, two of its members, namely ADAM10 and ADAM17, seem to be indispensable for development as judged by the complete embryonic or neonatal lethality of the knockout mice [1].

ADAM17 has been initially identified as the main sheddase responsible for releasing the soluble form of tumor necrosis factor (TNF) from the plasma membrane [2], [3]. To date, almost 80 substrates susceptible to ADAM17 proteolysis have already been recognized [4], [5]. The central, physiological role of ADAM17 in the shedding of TNF and both its receptors, several growth factors of the epidermal growth factor (EGF) family [transforming growth factor-α (TGFα), heparin binding EGF-like growth factor (HB-EGF), amphiregulin], E-selectin, fms-like tyrosine kinase receptor-3 ligand (Flt-3L), and platelet glycoprotein Ib alpha chain (GP1BA) has been confirmed *in vivo* through the use of genetically engineered mice [5]. Most of ADAM17 substrates can be classified into one of two functional groups: (***i***) growth factors and growth factor receptors, whose limited proteolysis constitutes the signal for proliferation, migration, and survival; (***ii***) cytokines, cytokine receptors, cell adhesion molecules, and other protein regulators of the immune system. In the case of the first group the activity of ADAM17 is always favorable for growth/survival/motility. As far as the second group is concerned the effect of ADAM17 is ambiguous: pro- as well as anti-inflammatory effects can be expected, although the opinion prevails that the former would dominate.

ADAM17 has been reported to be overexpressed in a number of different human tumors including brain, breast, colon, gastric, kidney, liver, lung, ovarian, pancreatic, and prostate (reviewed in [6]). In some cases, in which the exact pattern of ADAM17 expression within the tumor tissue has not been determined, it cannot be excluded that non-neoplastic cells, e.g. infiltrating macrophages, endothelial cells or stromal fibroblasts are the major source of ADAM17. However, some studies unambiguously documented increased ADAM17 expression within tumor cells and its correlation with tumor progression [7], [8], [9]. Well-designed experimental approaches confirmed the importance of ADAM17 in tumor progression. Overexpression of ADAM17 in MCF-7 breast cancer cells up-regulated, while ADAM17 silencing in MDA-MB-435 cell line down-regulated cell proliferation and invasiveness *in vitro* [10]. Targeting ADAM17 with siRNA reverted the malignant phenotype in T4-2 breast cancer cell line [11] and strongly inhibited the growth of two independent human renal carcinoma cell lines in immunocompromised mice [12].

The major mechanism by which ADAM17 contributes to tumor progression was proposed to involve the activation of growth factor receptors of EGFR (ErbB/HER) family. This activation, which stimulates multiple signaling pathways, leads to increased cell survival, proliferation, and motility. Simultaneous expression/overexpression of receptors belonging to EGFR family with the expression/overexpression of their ligands is often observed in cancers of epithelial origin and is responsible for the autocrine stimulation of their growth and development. The growth factors of EGF family are produced as transmembrane proteins (proproteins), but in general only their soluble forms, generated via shedding, are able to stimulate their receptors effectively [13]. Thus the activity of ADAM17, the major sheddase for TGFα, HB-EGF, and amphiregulin, may be regarded as prerequisite for the pro-tumorigenic effects of these growth factors. In fact, the requirement of ADAM17-mediated shedding of the mentioned growth factors for efficient EGFR activation was confirmed in numerous studies [12], [14], [15], [16], [17]. ADAM17 is also able to release ectodomains of other growth factors such as epiregulin, epigen, and neuregulin-1 (NRG-1) and along with other proteases may enable their signaling.

Tumor development depends not only on the tumorigenic potential of the cancer cells but also on the interactions between tumor cells and their environment, especially on the mutual relations between the tumor cells and the immune cells. As ADAM17 is a major sheddase for a number of protein regulators of the immune system, as well as for proteins involved in cell-cell and cell-matrix interactions, it is assumed that ADAM17 may affect tumor growth also via growth factor independent pathways [6], although at present this hypothesis lacks strong experimental support.

As we were looking for a suitable mouse model to study the effectiveness of potential ADAM17 inhibitors in cancer therapy, we coincidentally found a tumor cell line, namely MC38CEA colon carcinoma, in which ADAM17-silencing had no effect on *in vitro* cell proliferation but, despite that, strongly inhibited *in vivo* tumor growth. Therefore, the aim of our work is to elucidate novel pathways via which ADAM17 promotes tumor development. We have excluded the possibility that ADAM17 contributes to MC38CEA tumor progression via shedding of the ligands for EGFR and ErbB4. Our results suggest that NRG-1 released from MC38CEA by ADAM17 activates ErbB2 phosphorylation, which could play a role in an autocrine tumor promoting network. ADAM17-silencing resulted in decreased cell motility as well as expression of protumorigenic genes including those important for angiogenesis such as vascular endothelial growth factor-A (VEGF-A) and matrix metalloproteinase-9 (MMP-9). Additionally, we demonstrate that the decreased expression of ADAM17 in MC38CEA tumors influenced immune response and particularly affected the intratumoral cytokine profile including increased concentration of TNF and interferon-γ (IFNγ) that had strong synergistic proapoptotic effect towards cancer cells.

Our findings imply that at the cellular level ADAM17 may augment malignant potential of colon carcinoma cells by increasing their motility and expression of pro-angiogenic factors while at the tissue level enhances angiogenesis and affects the cross-talk between tumor cells and immune system.

## Materials and Methods

### Mice

Female C57BL/6 mice were purchased from the Mossakowski Medical Research Centre, Polish Academy of Sciences, Warsaw, and housed with ample access to food and water. They were used at 7–8 weeks of age. All experimental procedures were approved by the Animal Experiments I Local Ethics Committee, Kraków (Approval No 11/2007) and all efforts were made to minimize animal suffering.

### Cell culture

Murine colon adenocarcinoma MC38 [18], stably expressing human carcinoembrional antigen (MC38CEA) was a gift from Dr. Michał Bereta (Jagiellonian University, Kraków) [19]. The cells were cultured in DMEM (Lonza) supplemented with 10% heat-inactivated fetal calf serum (FCS) (Lonza) at standard conditions. Every few passages the culture medium was enriched in geneticin (1.5 mg/ml) and for mock-transfected and ADAM17-silenced MC38CEA (see below) additionally in hygromycin (400 µg/ml). The cell cultures were routinely tested by PCR for mycoplasma contamination using mycoplasma-specific primers.

### Generation of MC38CEA cell lines with stably silenced ADAM17

MC38CEA cells were transfected with pGeneClip™Vector (Promega) coding for ADAM17 shRNA or for non-interfering control shRNA using lipofectamine-2000 (Invitrogen Life Technologies) according to the manufacturer's protocol. Stably transfected cells were obtained by hygromycin selection. On the basis of our preliminary results three cell lines with the highest level of ADAM17-silencing (S2, S3, S35) and three control cell lines (mock-transfected; M1, M2, M3) were chosen for the experiments. The coding sequences of ADAM17 shRNA and control shRNA were AACGAATGCTGGTGTATAAGT and GCTCAGATATCCAGTCATGTT, respectively.

### TNF shedding assay

MC38CEA cells (5×10^5^) were incubated for 30 min in 100 µl of DMEM with PMA (100 ng/ml) or with corresponding volume of DMSO (solvent). The level of TNF released to the medium was evaluated using ELISA (R&D Systems).

### Neuregulin-1 shedding assay

MC38CEA cells were cultured for 24 h in 12-well plates (2.5×10^5^ cells/well) in DMEM containing 5% FCS and for the next 24 h in 0.5 ml of DMEM/F12 enriched in BSA (0.5 mg/ml), human holotransferrin (5 µg/ml) and sodium selenite (2 ng/ml) (complete DMEM/F12). Concentration of NRG-1 released to the medium was evaluated by ELISA (Uscn Life Science Inc.) according to the manufacturer's instruction.

### Analysis of VEGF-A secretion

MC38CEA cells were plated in 96-well plates (5×10^3^ cells/well). Next day medium was changed for DMEM containing 2% FCS and the cells were cultured for 48 h. Concentration of VEGF-A in the culture media was measured using mouse VEGF ELISA Duoset (R&D Systems) according to the manufacturer's protocol.

### Proliferation/viability assay (MTT test)

MC38CEA cells were plated in 96-well plates (5×10^3^ cells/well) and cultured for 5 days in DMEM containing 0.5% FCS or in DMEM without serum. Every day cell metabolic activity being an indicator of cell number and viability was measured using MTT assay [20]. The absorbance of solubilized formazan was measured at 545 nm.

### ^3^H-Thymidine incorporation

MC38CEA cells were cultured for 24 h in 12-well plates (2.5×10^5^ cells/well) in DMEM containing 5% FCS and for the next 24 h in fresh, serum-free DMEM or in DMEM containing 5% FCS. For the last 6 h of incubation, 1 µCi of ^3^H-thymidine was added to each well. After intensive washing with PBS, the cells were permeabilized with ice-cold methanol, washed again with PBS, and the radioactivity incorporated into DNA was determined by liquid scintillation counting in a Wallac β-counter (Perkin Elmer).

### Western blotting analysis of ErbB2 phosphorylation

MC38CEA cells (2.5×10^5^ cells/well) were incubated in complete DMEM/F12. The cells were lysed on ice in buffer containing 50 mM Tris-HCl pH 7.4, 150 mM NaCl, 0.1% SDS, 1% NP-40 and 1% CHAPS, Complete Protease Inhibitor Cocktail (Roche Applied Science) and additionally 1 mM PMSF, 5 mM NaF and 2 mM Na_3_VO_4_. Western blotting analysis was performed according to the standard protocol [21]. Membranes were probed with rabbit polyclonal anti-ErbB2 (phospho Y1248) antibody (Abcam) at 1 ∶1000 dilution and mouse monoclonal anti-GAPDH antibody clone 6C5 (Biodesign Int.) (0.5 µg/ml) and HRP-conjugated appropriate secondary antibodies. Bands were visualized using Immobilon Western Chemiluminescent HRP Substrate (Millipore). Quantification: Serial dilutions of a lysate obtained from the M1 cells incubated for 10 min with rmNRG-1 were subjected to Western blotting with anti-phosphorylated ErbB2 in order to find a slope and a range of linearity of the standard curve. The curve slope allowed to convert relative autoradiographic signals to relative levels of phosphorylated ErbB2 in the parallel experiments. For each sample, the autoradiographic signal of phosphorylated ErbB2 was normalized to the GAPDH signal. The values obtained for mock-transfected M1 cell line were taken as 100% in all performed experiments.

### Caspase activity assay

MC38CEA cells were cultured for 24 h in white-walled 96-well plates (5×10^3^ cells/well) in DMEM containing 5% FCS. Then the medium was changed for DMEM containing 2% FCS and the cells were incubated for 6 h with 120 µl of medium alone, or with recombinant human TNF (10 ng/ml), or with recombinant mouse IFNγ (10 ng/ml) or with both cytokines. The activity of caspases 3 and 7 in 30 µl samples of the media was evaluated using Caspase-Glo 3/7 Assay (Promega) according to the manufacturer's instruction. Chemiluminescence was measured using Infinite 200 PRO chemiluminometer (Tecan).

### Analysis of various mRNA levels using quantitative reverse-transcription PCR (qRT-PCR)

RNA was isolated from cells by guanidinium thiocyanate-phenol-chloroform extraction and 2 µg of each sample was used to synthesize cDNA using the reverse transcription kit (Promega). For obtaining ErbB4 cDNA the specific primer (5′-CTTTTTGATGCTCTTTCTTCTGAC-3′) apart from oligo(dT) was used. Samples of cDNA (∼5 ng) were amplified using one step real time PCR kit (Kapa Biosystems) and the RotorGene-3000 thermocycler (Corbett Research, Australia). Following primers (final concentration 200 nM) were used:

ADAM17 – F: CCAGGAGCGCAGCAACAAGGT, R: TCCTATCACTGCACTGCACACCCG; TNF – F: CATCTTCTCAAAATTCGAGTG, R: TGGGAGTAGACAAGGTACAA;

IFNγ – F: GCTTTGCAGCTCTTCCTCAT, R:GTCACCATCCTTTTGCCAGT;

MCP-1 – F: AGCACCAGCCAACTCTCACT, R: GCTGCTGGTGATCCTCTTGT;

VEGF-A – F: ATGCGGATCAAACCTCACCAAGGC, R: TTAACTCAAGCTGCCTCGCCTTGC;

TGFα – F: TGATCCACTGCTGTCAGCTC, R:CTTGGTTGGGCTGTCATCGG;

EGFR – F: ACACTGCTGGTGTTGCTGAC, R:CCCAAGGACCACTTCACAGT;

ErbB2 – F: AGGGGCTGGCTCCGATGTGTTT, R: GGCTGGTTCACATACTCGGGCT;

ErbB3 – F: CCACGTGGGAGCCAGAGTCTTT, R: TTCAGCCTCAGAGCCCGTCACA;

ErbB4 – F: GAAATGTCCAGATGGCCTACAGGG, R: CTTTTTGATGCTCTTTCTTCTGAC;

EF2 – F: GACATCACCAAGGGTGTGCAG, R: GCGGTCAGCACAATGGCATA.

The levels of various mRNA in individual cell lines (mock-transfected and ADAM17-silenced were compared to that in wild type MC38CEA using the “delta delta Ct” relative quantitation method (Applied Biosystems). EF2 cDNA was used as reference.

### Time-lapse monitoring of individual cell movement

MC38CEA cells (1.5×10^5^ cells) were plated in a T25 culture flask in DMEM containing 10% FCS. After 24 h cell movement was recorded for 8 h at 5 min intervals using Leica DM IRE 2 microscope equipped with FW4000 software. The trajectories of 50 individual, randomly chosen cells were analyzed as previously described [22] in order to obtain: the total length of cell trajectory (TLT), the velocity of cell movement defined as a total length of cell trajectory/time of recording, the total length of displacement (TLD) and the coefficient of movement efficiency (CME) defined as the ratio of total length of cell displacement to total length of cell trajectory (TLD/TLT).

### Wound healing assay

MC38CEA cells (2×10^5^) were grown in a 6-well plate in DMEM containing 10% FCS until they reached monolayer. The medium was aspirated, a scratch wound was made across each well using a tip, monolayers were washed to remove detached cells and the cells were incubated in fresh, serum-free DMEM for 24 h. The pictures were taken at time 0 and 24 h after wounding, and the widths of the wounds were measured. In some experiments the cells were starved for 3 h before wounding and then wounded monolayers were incubated in serum-free DMEM alone or in the medium enriched in rmNRG-1. The generation and testing of rmNRG-1 is described in Supporting Information. The lengths of cell displacement are expressed as the difference between average pre- and post-healing wound widths. The average width was calculated on the basis of 5 measurements at random positions along each wound.

### *In vivo* tumor growth

MC38CEA cells (ADAM17-silenced, mock-transfected and wild type) were trypsinized and washed twice with PBS. 5×10^5^ cells in 100 µl of PBS were injected subcutaneously into one flank of each mouse. Tumor sizes were evaluated by caliper every 2–3 days and the tumor area was determined by multiplying measurements of two perpendicular diameters.

### Cytometric bead array

The levels of cytokines [interleukin-6 (IL-6), IL-10, monocyte chemoattractant protein 1 (MCP-1), IFNγ, TNF, and IL-12p70] were measured in serum and tumor lysates using the BD Cytometric Bead Array (CBA) Mouse Inflammation Kit (Becton Dickinson), according to the manufacturer's instruction using 50 µl of serum or 100 µg of total lysate protein obtained using RIPA buffer (25 mM Tris-HCl, pH 7.6, 150 mM NaCl, 1% NP-40, Complete Protease Inhibitor Cocktail). The fluorescence of samples was acquired on the LSRII flow cytometer (Becton Dickinson) and analyzed using Becton Dickinson FCAP Array™ Software.

### Immunohistochemical staining of blood- and lymphatic vessels

Immunochemical staining of frozen tissue sections was performed according to the standard protocol [23]. Briefly, 10 µm-thick cryosections were air-dried and fixed with a zinc-based fixative for 24 h, blocked with 10% donkey- and 10% goat serum in PBS and stained with (*i*) hamster anti-CD31/Alexa fluor 546-conjugated anti-hamster IgG; (*ii*) rabbit anti-lymphatic endothelium specific antigen-1 (LYVE-1)/Alexa Fluor 488-conjugated anti-rabbit IgG; (*iii*) rat anti-CD11b/Alexa fluor 594-conjugated anti-rat IgG; *(iv)* APC-conjugated rat anti-CD45. In negative controls primary antibodies were omitted. Cell nuclei were counterstained with DAPI. Sections were imaged on a Zeiss LSM 510 Meta confocal microscope at magnification 400×. Five randomly chosen fields of peritumoral area and five randomly chosen fields of tumor peripheral area were analyzed in each section. The area covered by CD31^+^LYVE-1^−^ blood vessels in tumor periphery were calculated using MetaMorph imaging software and expressed as the percentage of the total area.

### Zymography

Zymography was performed according to a standard protocol. Cells were cultured for 24 h in 6-well plates (5×10^5^ cells/well) in DMEM containing 5% FCS and for the next 24 h in fresh, serum-free DMEM. The samples of cell media (20 µl) were subjected to SDS-PAGE in 10% gels containing 0.1% gelatin. The gels were stained with Coomassie Brilliant Blue, resulting in a blue background of nondegraded gelatin with cleared bands of proteolytic activity.

### Statistical analysis

Statistical analysis was performed using the Student's *t*-test, with *P*<0.05 being considered significant.

## Results

### Generation of ADAM17-silenced MC38CEA cell lines

**Figure 1:**
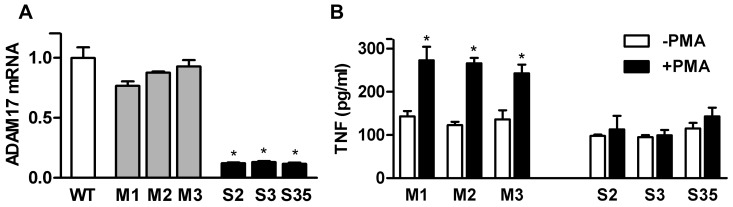
Analysis of ADAM17-silencing in MC38CEA cells. (**A**) Quantitative RT-PCR analysis of ADAM17 mRNA levels in the wild-type MC38CEA (WT) and in the MC38CEA cell lines stably transfected with control shRNA (M1, M2, M3), or with ADAM17 shRNAs (S2, S3, S35). * – *P*<0.01 *vs.* WT and each M cell line. (**B**) The levels of TNF released to the medium from mock-transfected or ADAM17-silenced MC38CEA cell lines incubated in the absence or presence of PMA, measured by ELISA. Bars represent average values ± SD from 3 independent experiments performed in duplicates. * – *P*<0.01 *vs.* unstimulated cells.

In order to study the potential role of ADAM17 in the growth and progression of colon tumors we generated several MC38CEA colon carcinoma cell lines with stably silenced ADAM17 expression via transfection with ADAM17 shRNA coding vector. Parallelly, we obtained MC38CEA cell lines stably transfected with the equivalent vector coding for non-interfering control RNA. In our experiments we used three mock-transfected cell lines (M1, M2, and M3) that showed ADAM17 mRNA levels similar to that of the wild-type cells (WT) and three cell lines with strong (around 80–88%) silencing of ADAM17 (S2, S3, S35) (Fig. 1A). All generated MC38CEA cell lines (mock-transfected and silenced) expressed TNF mRNA at relatively low but similar levels (data not shown). ADAM17 activity was estimated in all the cell lines by measuring the concentration of TNF released to the medium from the cells in response to PMA-treatment. As expected, this ADAM17-mediated process was strongly inhibited in ADAM17-silenced cells (Fig. 1B). Thus, the resulting cell lines meet the basic criteria for a proper model to study the role of ADAM17 in tumor progression.

### ADAM17-silencing inhibits *in vivo* growth of MC38CEA tumors

**Figure 2:**
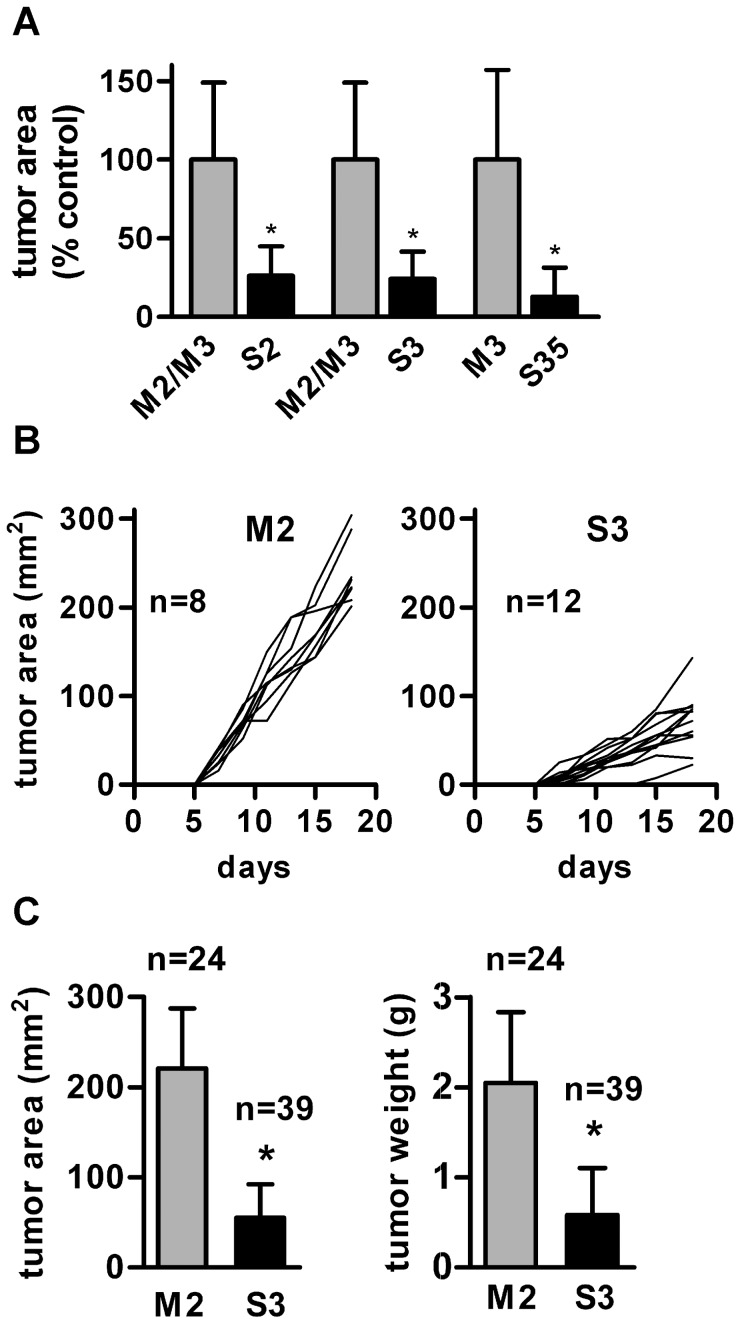
Effect of ADAM17-silencing on MC38CEA tumor growth. (**A**) Comparison of the final areas of different mock-transfected and ADAM17-silenced MC38CEA tumors. Data are from one (for S35) or two experiments (for S2 and S3) and at least 5 mice per data point. Control mice were injected with M2 (5 mice) or M3 (5 mice) cells. As there were no statistically significant differences between the areas of M2 and M3 tumors, the average areas of mock-transfected tumors (of both M2 and M3 in experiments 1 and 2 and M3 in experiment 3) were taken as 100%. (**B**) Individual tumor growth curves from one representative experiment. Mice were injected with mock-transfected (M2) or ADAM17-silenced MC38CEA (S3) cells and the tumor areas were measured every 2–3 days. (**C**) Overall data from 5 independent experiments presenting the final areas and the final volumes ± SD of mock-transfected (M2) or ADAM17-silenced MC38CEA (S3) tumors. * – *P*<0.01 *vs.* mock-transfected tumors.

We next decided to evaluate the effect of ADAM17-silencing on *in vivo* growth of MC38CEA tumors. After confirming that the mock-transfection does not influence *in vivo* MC38CEA tumor development (data not shown) we compared the growth of tumors induced by implanting the same number (5×10^5^) of cells of two different mock-transfected cell lines and three different ADAM17-silenced cell lines. As shown in Fig. 2A all ADAM17-silenced tumors at the end of the experiment were over 70% smaller than the mock-transfected ones. Because individual mock-transfected and ADAM17-silenced cell lines differed in the level of CEA expression, we performed the majority of the following experiments using M2 and S3 cell lines which expressed almost identical levels of CEA as measured by flow cytometry (data not shown) to ensure correspondence between the two model cell lines. Figure 2B presents growth curves of tumors in individual mice. The measurable tumors appeared on day 6 or 7 in case of mock-transfected MC38CEA and usually one day later in case of ADAM17-silenced cells. The rate of the tumor growth was constant for each cell line throughout the duration of the experiment though significantly lower for ADAM17-silenced tumors. Overall results of several experiments confirmed that ADAM17-silenced tumors, as estimated both by the surfaces of their outer parts and the weights of tumors isolated at the end of the experiments, were significantly smaller than mock-transfected ones (Fig. 2C). At the end of some experiments, the cells isolated from tumors were cultured for two weeks (in the absence of selection antibiotic) in order to remove immune cells and then the maintenance of ADAM17 silencing in MC38CEA cells was confirmed by quantitative RT-PCR (data not shown). The results indicate that ADAM17 promotes development of MC38CEA tumors.

### ADAM17 silencing does not affect MC38CEA growth *in vitro*

In some tumor models ADAM17 was shown to support autocrine stimulation of cell growth by shedding EGF family members. In those models, expression of ADAM17 positively correlated with *in vivo* tumor progression and *in vitro* proliferation rate of tumor cells in serum-free medium [11], [12]. Does the same mechanism explain the requirement of ADAM17 for a robust MC38CEA development?

**Figure 3:**
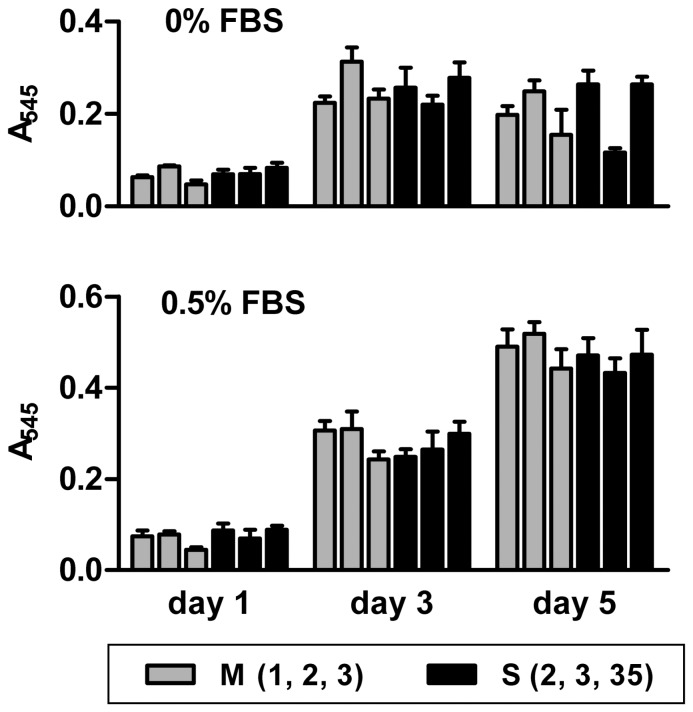
Effect of ADAM17 silencing on proliferation and viability of MC38CEA cells. The cells were grown in serum-free or serum-low DMEM and their growth and viability were monitored daily using the MTT assay. Bars represent average values ± SD from 3 independent experiments performed in sextuplicates.

Surprisingly, the silencing of ADAM17 in MC38CEA cells did not affect *in vitro* cell growth and viability. All mock-transfected and ADAM17-silenced cell lines showed exactly the same ^3^H-thymidine incorporation (407±16 cpm/10^6^ cells) during 6-h incubation with the radiolabeled nucleotide that followed a 24-h starvation period (0% FBS). In another type of experiment the cells were plated at a low density and cultured for 5 days in the absence of serum or at low serum concentration (0.5%). The cells completely deprived of serum stopped growing no later than on the third day after plating, which is in contrast to many cancer cell lines that continue growth upon serum deprivation [12], [24], [25], and indicates that autocrine stimulation does not play a major role in growth of MC38CEA cells in culture. Moreover, in both cases (0% and 0.5% serum) the total mitochondrial activity, which is indicative of the number of viable cells, was the same for all mock-transfected and ADAM17-silenced cell lines after 1, 3, and 5 days in culture, although deleterious effects of the lack of serum were evident in some mock-transfected as well as ADAM17-silenced cultures on day 5 (Fig. 3). The results indicate that ADAM17 does not influence proliferation of MC38CEA cells *in vitro*, and suggest that ADAM17 does not promote tumor development via activation of growth factors.

### MC38CEA cells do not express EGFR or ErbB4

**Figure 4:**
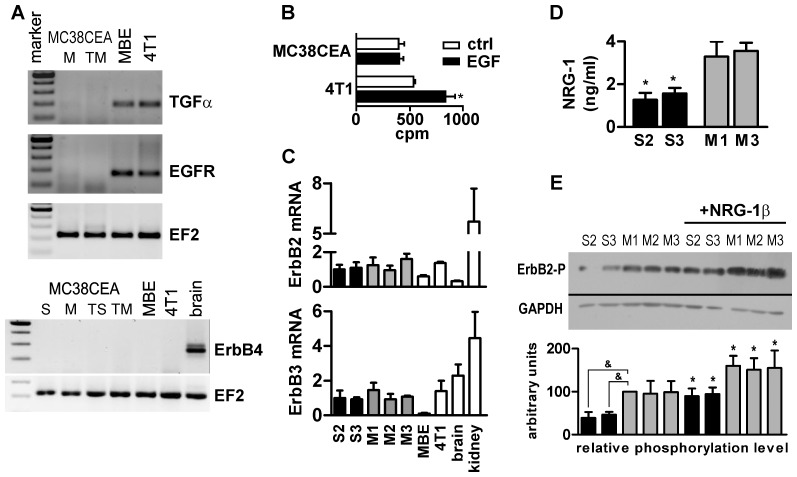
Expression of growth factors and growth factor receptors by MC38CEA colon carcinoma. (**A**) RT-PCR analysis of the expression of TGFα, EGFR and ErbB4 in MC38CEA cells in culture, in MC38CEA tumors and in positive controls: murine brain endothelial cells (MBE) and murine breast cancer cell line (4T1) and murine brain. cDNA from: M – mock-transfected cells, TM – mock-transfected tumors, S – ADAM17-silenced cells, TS – ADAM17-silenced tumors. Amplification of cDNA coding for elongation factor 2 (EF2) was performed as a control of samples' quality. (**B**) Analysis of the effect of exogenous EGF on ^3^H-thymidine incorporation into MC38CEA or 4T1 cells (positive control). * – *P*<0.01 *vs.* control. (**C**) Quantitative RT-PCR analysis of ErbB2 and ErbB3 expression in ADAM17-silenced (S2, S3) and mock-transfected (M1, M2, M3) cell lines and in control cell lines, MBE and 4T1 as well as in murine tissues. (**D**) The levels of NRG-1 released to the medium from ADAM17-silenced (S2, S3) and mock-transfected (M1, M3) cells measured by ELISA. * – *P*<0.01 *vs.* M1 and M3. (**E**) Western blotting analysis of ErbB2 phosphorylation in ADAM17-silenced (S2, S3), mock-transfected (M1, M2, M3) and wild-type (wt) MC38CEA cells incubated in the serum-free medium alone or in the medium enriched in exogenous rmNRG-1β. GAPDH was used as a loading control. Quantification of Western blot signals is described in Materials and Methods and is based on at least 3 independent experiments for a given cell line. * – *P*<0.01 *vs.* the same cell line incubated w/o rmNRG-1β; & – *P*<0.001 *vs.* M1. All data in panels A, B, C and D come from at least 3 independent experiments performed in duplicates.

ADAM17-silencing was reported previously to decrease cell proliferation. We did not observe this effect in MC38CEA cells and so we tested which of the signaling components were missing. The expression of different members of EGF family and EGF receptor family was analyzed in MC38CEA cells at the mRNA level. We found that the cells did not express TGFα for which ADAM17 is the predominant sheddase (Fig. 4A). HB-EGF, although visible at the mRNA level, was not detectable at the protein level (data not shown). What is more, MC38CEA cells did not express either EGFR or ErbB4, the receptors that may respond to stimulation by TGFα, HB-EGF, and some other factors of EGF family. Moreover, the proliferation of MC38CEA was not stimulated by external EGF in contrast to control 4T1 cells (Fig. 4B). Thus we ruled out the possibility that ADAM17 promoted MC38CEA tumor growth through the activation of the growth factors for which it was identified as the main sheddase.

### MC38CEA cells express ErbB2, ErbB3, and their ligand NRG-1

The family of EGF receptors also contains ErbB2 and ErbB3 which may form heterodimers and generate intracellular signal in response to neuregulins (NRG) 1 and 2. NRG-1 has been shown to be susceptible to proteolysis by various ADAMs including ADAM17 [26], [27], [28], [29]. Therefore, we analyzed the expression of ErbB2, ErbB3, and NRG-1 in MC38CEA cells and found out that the cells, as measured at the mRNA level, expressed both ErbB2 and ErbB3 (Fig. 4C). Moreover, not only did the cells release considerable quantities of NRG-1 to the culture medium, but also the mock-transfected cells released more than twice as much NRG-1 as ADAM17-silenced cells (Fig. 4D). In spite of this, we did not observe any autocrine growth stimulation in MC38CEA cultures nor any difference in the proliferation rate between mock-transfected and ADAM17-silenced cells. Exogenous rmNRG-1β did not affect growth of these cells either (data not shown). Since the ErbB2/ErbB3 phosphorylation is prerequisite for intracellular signaling, we analyzed the level of ErbB2 phosphorylation to determine whether in MC38CEA, NRG-1 may activate the receptor in an autocrine manner. Western blotting analysis revealed that ADAM17-silencing resulted in a decreased level of ErbB2 phosphorylation (Fig. 4E). Moreover, ADAM17-silenced cells retained sensitivity to NRG-1, as exogenous NRG-1β caused phosphorylation of ErbB2 to the level comparable with that observed for the mock-transfected cells (Fig. 4E). However, even at a very high concentration of rmNRG-1β (100 ng/ml), the level of ErbB2 phosphorylation in ADAM17-silenced cells did not reach the level of its phosphorylation in mock-transfected ones suggesting a diminished sensitivity of ADAM17-silenced cells to NRG-1.

### ADAM17-silencing decreases MC38CEA motility *in vitro*

**Figure 5:**
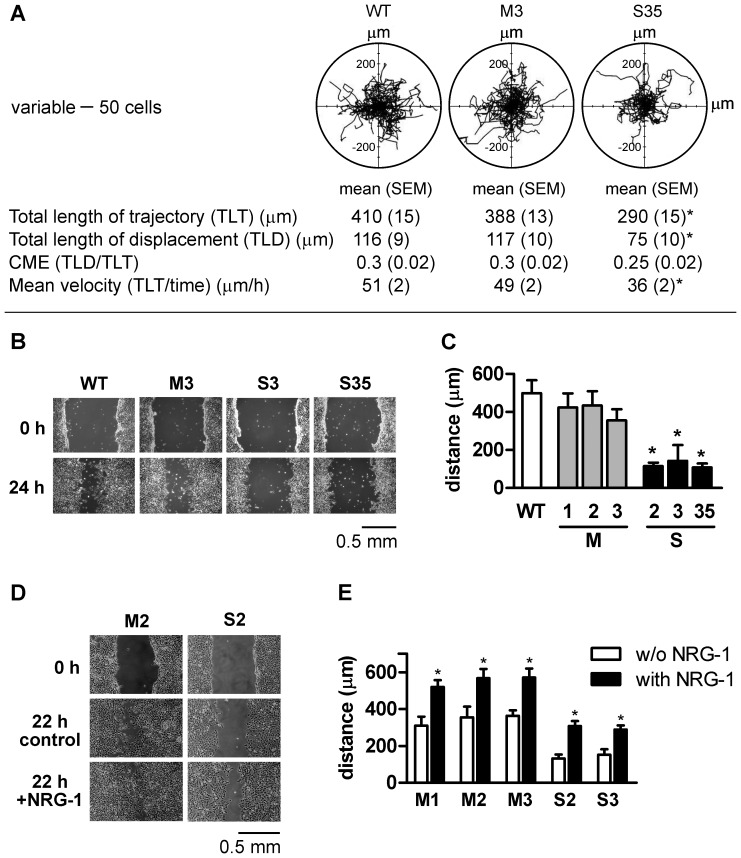
Effect of ADAM17 silencing on cell motility. (**A**) Exemplary trajectories of migrating wild type (WT), mock-transfected (M3) and ADAM17-silenced (S35) MC38CEA cells displayed in circular diagrams drawn with the initial point of each trajectory placed at the origin of the plot. Each panel shows the trajectories of 50 cells. The variables of cell movement are presented below diagrams. CME, coefficient of movement efficiency; *P<0.05 (*vs.* wild type and mock-transfected cells). Data are from one representative experiment. Comparable results were obtained in the second experiment with M3 and S35 as well as in two experiments, in which the movements of WT, M2, and S3 were analyzed. (**B, C**) Wound healing assay (**B**) Representative wound images. Micrographs taken at 0 and 24 h after scratch wounding of cell monolayers. (**C**) Quantification of the results of two independent experiments performed in duplicates for each cell line. The lengths of cell displacement are expressed as a difference between average pre- and post-healing wound. * – *P*<0.01 *vs.* M3. (**D**) Influence of rmNRG-1β on mock-transfected- and ADAM17-silenced MC38CEA cells – representative wound images. Wounded monolayers were incubated for 22 h in serum-free medium in the absence (control) or in the presence of rmNRG-1β. (**E**) Quantification of the results from a single experiment representative of three performed. * – *P*<0.01 *vs.* the same cell line incubated w/o rmNRG-1β.

To evaluate a potential impact of ADAM17 silencing on the cell motile activity we analyzed the movement of individual cells of WT, mock-transfected, and ADAM17-silenced lines recorded during an 8-h period. The trajectories of the cells are shown as circular diagrams in Fig. 5A. These analyses indicated that the lengths of trajectories and consequently the mean velocities of movement as well as lengths of cell displacement were comparable between wild-type and mock-transfected cells and significantly lower in ADAM17-silenced cells (Fig. 5A). Low CME values, similar for all three cell types, indicate randomness of the cell movement.

The importance of ADAM17 for MC38CEA cell motility was further confirmed in a wound healing assay. ADAM17-silenced cells migrated over a much shorter distance to cover a gap in the cell monolayer than the wild-type and mock-transfected cells (Fig. 5B, C).

Most reports which demonstrate ADAM17-mediated stimulation of cell migration also indicate growth factor-dependent mechanisms [12], [16], [30]. Hence the question arose whether the observed difference in NRG-1 shedding may translate into a difference in cell motility of MC38CEA cells. Addition of exogenous rmNRG-1β to wounded monolayers resulted in the stimulation of migration of both mock-transfected and ADAM17-silenced cells (Fig. 5D, E). However, ADAM17-silenced cells always migrated over a shorter distance than mock-transfected cells, even at a high concentration of NRG-1 (100 ng/ml). This effect of exogenous rmNRG-1β on cell motility perfectly reflects its effect on phosphorylation of ErbB2 in mock-transfected and ADAM17-silenced MC38CEA cells and suggests that although NRG-1 may add to the migratory potential of MC38CEA cells, inhibition of its shedding is not the only phenomenon responsible for diminished motility of ADAM17-silenced cells.

### Tumors of mock-transfected and ADAM17-silenced MC38CEA differ in cytokine profiles

**Figure 6:**
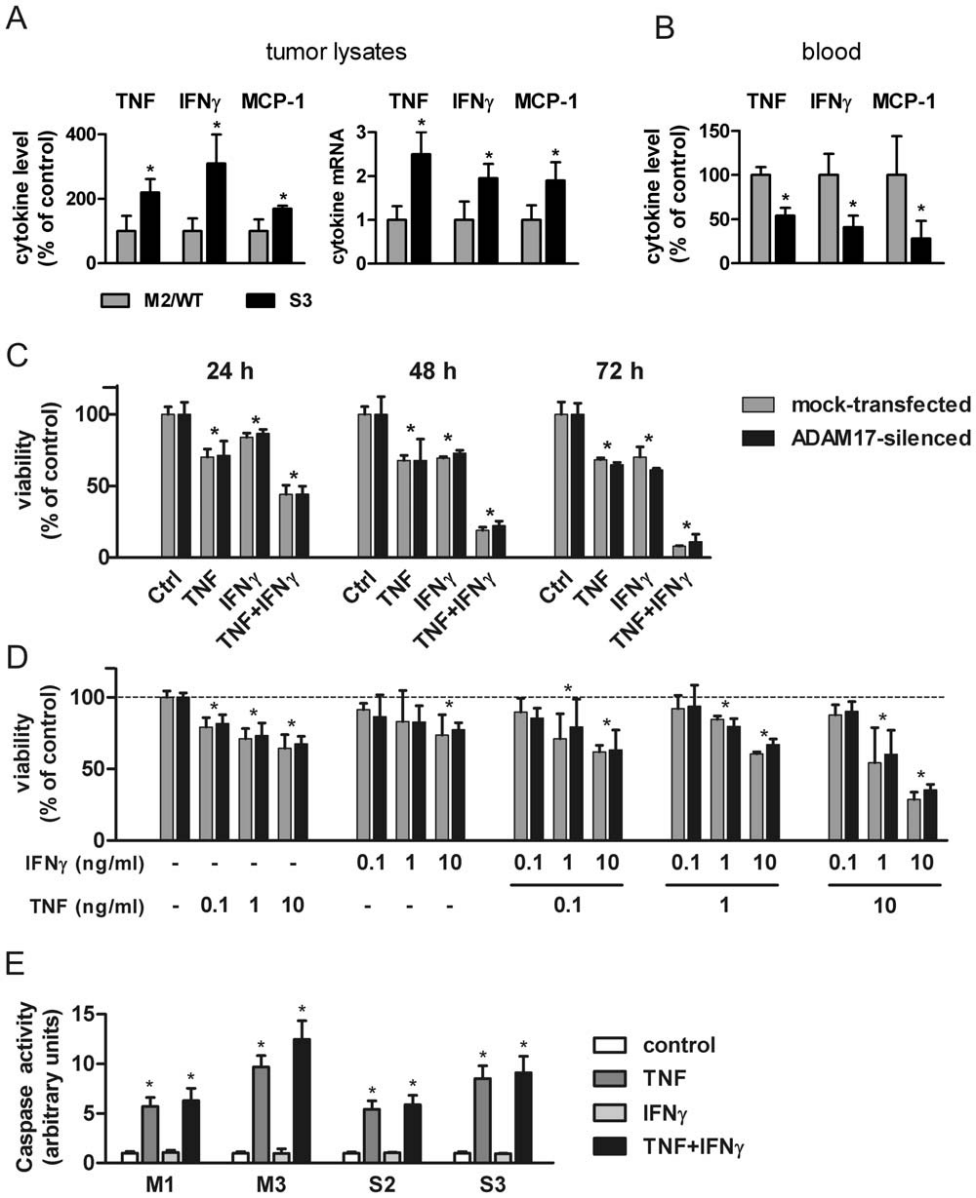
Analysis of the cytokine profiles in MC38CEA tumors and their cytotoxic effects on MC38CEA cells. (**A**) Relative protein and transcript levels of TNF, IFNγ and MCP-1 in lysates from wild type (WT), mock-transfected (M2) and ADAM17-silenced (S3) MC38CEA tumors measured by cytometric bead array immunoassay (CBA) or qRT-PCR, respectively. Average levels of each cytokine or its mRNA in M2 and/or WT tumor lysates were taken as 100% (CBA) or 1 (qRT-PCR). Control grups were as follows: M2 (three experiments, 5 mice per each experiment), WT (5 mice in one experiment) and both M2 (4 mice) and WT (5 mice) in one experiment. (**B**) Relative levels of TNF, IFNγ, and MCP-1 in sera from mice bearing WT, mock-transfected (M2), and ADAM17-silenced (S3) MC38CEA tumors at the end of the experiments. (**C, D**) The influence of TNF and IFNγ on MC38CEA viability. (**C**) Mock-transfected and ADAM17-silenced MC38CEA cells were incubated in DMEM/2% FCS with TNF (10 ng/ml) or IFNγ (50 ng/ml) or with TNF+IFNγ for 24, 48 or 72 h and then cell viability was assayed using MTT test. (**D**) Mock-transfected and ADAM17-silenced MC38CEA cells were incubated for 48 h in DMEM/2% FCS with TNF or IFNγ or with TNF+IFNγ at various concentrations and then cell viability was assayed using MTT test. (**E**) The influence of TNF and IFNγ on caspase activity in mock-transfected (M1 and M3) and ADAM17-silenced (S2 and S3) MC38CEA cells. Bars represent average values ± SD—for (**A**) and (**B**) from 5 independent experiments with at least 5 mice per each experimental group, * – *P*<0.05 *vs.* M2 and WT controls; for (**C**) and (**D**) from 3 independent experiments performed in triplicates, * – *P*<0.05 *vs.* untreated controls; (**E**) from 4 independent experiments performed in sextuplicates, * – *P*<0.01 *vs.* untreated controls.

We hypothesized that the immune system that contributes to tumor microenvironment is at least partially responsible for the inhibition of the growth of ADAM17-silenced tumors. To evaluate this hypothesis we have measured the concentration of selected cytokines: TNF, IFNγ, IL-12 (p70), MCP-1, IL-6, and IL-10 in the tumor lysates and in the sera taken from mice at the time of their sacrifice. The levels of IL-10 and IL-12 (p70) in tumor lysates and in sera of all the mice were below or at the detection limit in the majority of the experiments and, if measurable, no significant difference appeared between experimental groups (data not shown). Similarly, we did not observe statistically valid differences in the concentrations of IL-6 in the lysates from mock-transfected and ADAM17-silenced tumors (data not shown). However, the concentrations of three other cytokines: TNF, IFNγ, and MCP-1 were consistently and significantly higher in ADAM17-silenced tumors than in mock-transfected ones (Fig. 6A). Augmented levels of these cytokines resulted from their increased expression within tumors as they were accompanied by elevated levels of their transcripts in tumor lysates. At the same time, serum levels of TNF, IFNγ, and MCP-1 were lower in mice bearing ADAM17-silenced tumors than mock-transfected ones, which is not surprising as serum levels of the cytokines usually correlate with the tumor size (Fig. 6B). The cytokine concentrations varied between experiments and their ranges (for ADAM17-silenced tumors) are presented in Table 1.

**Table 1:** Ranges of the cytokine concentration between experiments.

	Serum (pg/ml)*	Tumor lysates (pg/mg protein)*
TNF	6.21–15.81	5.73–34.03
IFNγ	1.01–1.74	7.67–17.34
MCP-1	60.5–120.09	769.97–3164.45

* The values represent the lowest average and the highest average cytokine concentrations estimated in ADAM17-silenced tumor lysates and in sera from mice bearing ADAM17-silenced tumor taken from three independent experiment (at least 5 mice per group). Concentrations of cytokines in the sera of control healthy mice (pg/ml): TNF – 5.0±1.4; IFNγ – 1.5±0.5; MCP-1 – 20.6±4.6.

MC38CEA cells express *in vitro* abundant quantities of MCP-1 (∼1800 pg/ml/24 h/10^5^ cells) but we did not observe any significant differences in MCP-1 mRNA level and the level of MCP-1 released to the culture media between mock-transfected and ADAM17-silenced cells used in *in vivo* experiments, namely S3 and M2 (data not shown).

Moreover, MC38CEA cells express scarce amounts of TNF (∼4.5 pg/ml/24 h/10^5^ cells), but they do not produce IFNγ. An increased level of TNF protein in ADAM17-silenced tumors may partially result from a diminished activity of ADAM17, which prevents TNF release from tumor cells. However, increased levels of TNF mRNA in ADAM17-silenced tumors, the presence of immune cells in the tumor mass and a much lower expression of TNF in MC38CEA cells than that in macrophages prompt us to believe that the majority of TNF as well as all IFNγ in tumors originate from infiltrating immune cells.

### TNF and IFNγ synergistically affect viability of MC38CEA cells

We went on to analyze the influence of TNF and IFNγ on the viability of mock-transfected and ADAM17-silenced MC38CEA cells (Fig. 6C, D). Incubation of MC38CEA cells either with TNF or IFNγ resulted in a significantly diminished number of viable MC38CEA cells and, what is more, the cytokines showed a strong synergistic effect. Only around 10% of the cells treated simultaneously with high doses of both cytokines were still viable after 72 h, and both mock-transfected and ADAM17-silenced cells showed similar sensitivity to TNF+IFNγ mixture (Fig. 6C). Experiments in which the cells were exposed to various concentrations of TNF+IFNγ confirmed that the sensitivity of MC38CEA to these cytokines does not depend on ADAM17 level (Fig. 6D). Strong activation of effector caspases 3 and 7 in both cell types (Fig. 6E) indicates that TNF-induced apoptosis is an important element of the TNF+IFNγ-evoked MC38CEA cell death. Higher concentration of TNF and IFNγ in ADAM17-silenced than in mock-transfected tumors together with dose-dependent sensitivity of MC38CEA cells to these cytokines suggest that the slower growth of ADAM17-silenced tumors may at least partially result from their cytotoxic/cytostatic effects.

### ADAM17 silencing affects pro-angiogenic potential of MC38CEA cells

**Figure 7:**
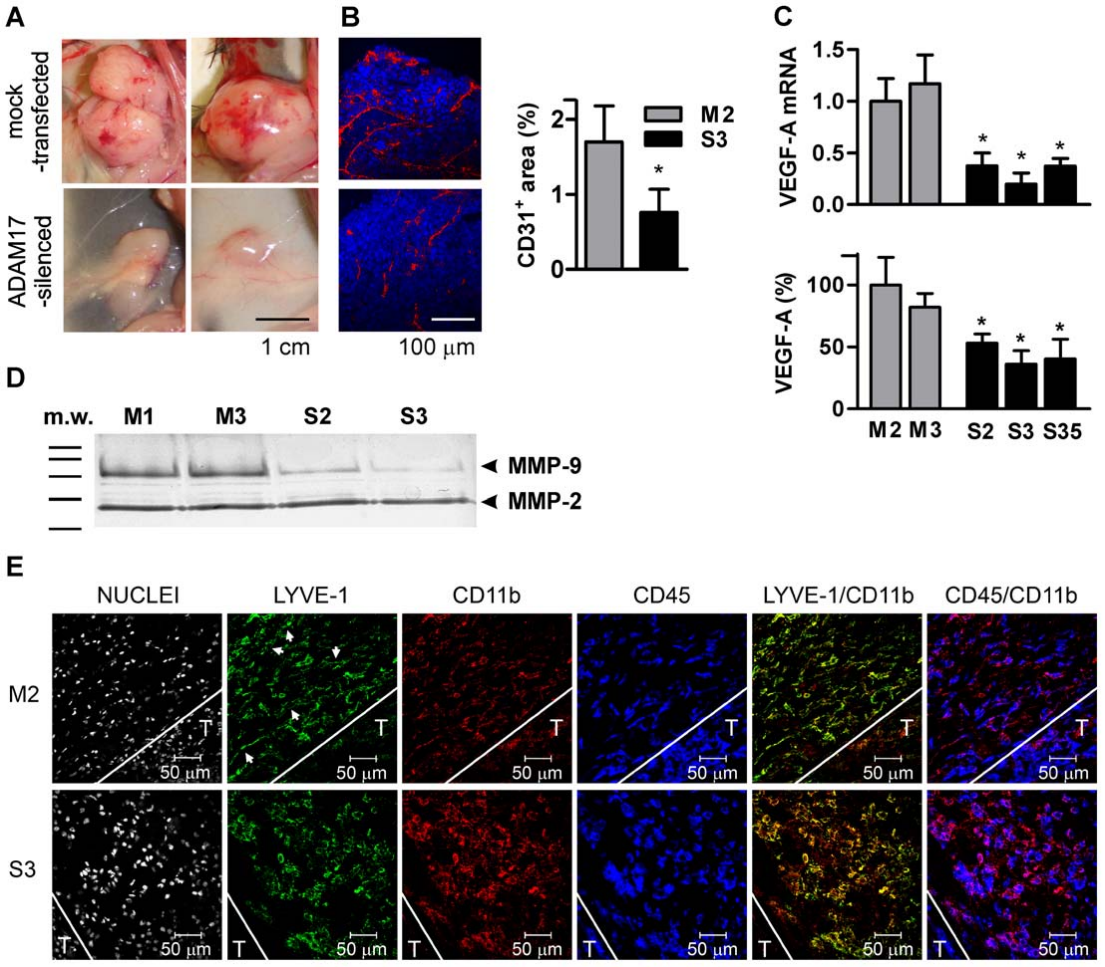
The effect of ADAM17-silencing on tumor angiogenesis and lymphangiogenesis. (**A**) Representative images of mock-transfected and ADAM17-silenced tumors on the 17^th^ day after s.c. injection of tumor cells. (**B**) Representative micrographs of peripheral tumor tissues and measurements of the areas covered by CD31-positive cells. Nuclei stained with DAPI (blue) and blood vessel endothelial cells stained with anti-CD31 (red). Experiment was performed twice with 5 tumors of each type analyzed per experiment. Five random fields were analyzed for each tumor section. * – *P*<0.01 *vs* M2. (**C**) Relative levels of VEGF-A mRNA analyzed by qRT-PCR and relative VEGF-A concentration in the culture media of mock-transfected and ADAM17-silenced MC38CEA cell lines. The average level of VEGF-A mRNA in mock-transfected (M2) cells was taken as 1 and the average concentration of VEGF-A in M2 culture medium was taken as 100%. Bars represent average values ± SD from 4 independent experiments performed in duplicates. * – *P*<0.01 *vs* M2 and M3. (**D**) Zymographic analysis of gelatinase activity in the culture media of mock-transfected (M1 and M3) and ADAM17-silenced (S2 and S3) MC38CEA cell lines. The result (shown as a photographic negative) is representative of 4 performed analyses. (**E**) Immunochemical analysis of lymphangiogenesis in peritumoral areas of mock-transfected and ADAM17-silenced MC38CEA tumors on the 17^th^ day after s.c. injection of tumor cells. The representative sections were stained with DAPI to visualize nuclei and immunostained with anti-LYVE-1, anti-CD45, and anti-CD11b antibodies. Single images and overlays of LYVE-1/CD11b and CD45/CD11b are presented. White lines indicate arbitrarily recognized borders between tumors (denoted as T) and peritumoral areas. Arrows indicate exemplary elongated mock-transfected LYVE-1^+^ cells arranged in a regular pattern.

The main difference between mock-transfected and ADAM17-silenced tumors, visible to the naked eye, was the density and organization of blood vessels on the tumor surface and in the dermis surrounding the tumors. Irregular, abnormally dilated, and hemorrhagic vessels visible in mock-transfected but not in ADAM17-silenced tumors indicate intense angiogenesis occurring in tumors with intact ADAM17 expression (Fig. 7A). To verify this finding at a microscopic level we stained tumor sections for CD31 and for lymphatic endothelium specific antigen-1 (LYVE-1) to compare the density of blood vessels (CD31^+^, LYVE-1^−^) in both types of tumors (Fig. 7B). LYVE-1-positive cells were present in peritumoral areas but not in tumors, therefore all CD31^+^ structures observed within tumor sections represented the blood vessels. The areas covered by blood vessels in peripheral parts of mock-transfected tumors were more than twice as big as the ones in peripheral parts of ADAM17-silenced tumors.

The question arose whether ADAM17-silencing inhibits tumor growth via inhibiting angiogenesis or, alternatively, whether slower growth of tumors delays occurrence of hypoxia and initiation of angiogenesis. The former hypothesis was supported by the observation that MC38CEA cells expressed *in vitro* VEGF-A, the major pro-angiogenic factor. After 48 h of culture of wild-type or mock-transfected MC38CEA (1×10^4^/100 µl), the concentration of VEGF-A in the culture medium reached 50 pg/ml. Moreover, the expression of VEGF-A was significantly higher in mock-transfected than in ADAM17-silenced cell lines, at both the transcript and the protein levels (Fig. 7C). Both cell types produced the same amount of another pro-angiogenic cytokine, namely KC (mouse functional homolog of IL8; data not shown), but secreted different amounts of matrix metalloprotease-9 (MMP-9) which is able to degrade collagen IV of the basement membrane and extracellular matrix and may therefore promote angiogenesis. As evaluated by zymography, the activity of MMP-9 was significantly higher in the media of mock-transfected than in the media of ADAM17-silenced cells cultured at the same density (Fig. 7D). Higher enzymatic activity of MMP-9 in mock-transfected cells correlated with a higher level of MMP-9 mRNA in these cells as evaluated by qRT-PCR (data not shown).

During the analysis of tumor angiogenesis we performed immunostaining using anti-LYVE-1 antibody in order to distinguish between blood vessels (LYVE-1^−^) and lymphatic vessels (LYVE-1^+^). This led to an interesting observation. The peritumoral areas of both mock-transfected and ADAM17-silenced MC38CEA tumors were infiltrated by CD45^+^CD11b^+^ cells that co-expressed the lymphatic endothelial marker LYVE-1 (Fig. 7E). Those cells did not penetrate tumor tissue. Although the numbers of the CD45^+^CD11b^+^LYVE-1^+^ cells seemed to be similar in the vicinity of both tumor types, their shape and arrangement were strikingly different. In the peritumoral area of ADAM17-silenced tumors the cells were round and evenly dispersed, while in the peritumoral area of mock-transfected tumors CD45^+^CD11b^+^LYVE-1^+^ cells were elongated and arranged in a more regular pattern. Drawing on the data published by Maruyama et al. who showed that macrophages can transdifferentiate into lymphatic endothelial cells that integrate into existing lymph vessels [31], we speculate that CD45^+^CD11b^+^LYVE-1^+^ observed in peritumoral area of MC38CEA tumors represent the type of transdifferentiating macrophages that would contribute to the formation of new lymphatic vessels. It seems that this process is delayed in case of ADAM17-silenced tumors.

## Discussion

### ADAM17 promotes MC38CEA tumor development in the absence of EGFR and ErbB4 signaling

ADAM17 is a predominant sheddase for a number of growth factors of EGF family including TGFα, HB-EGF and amphiregulin [32], [33]. In many tumors overexpression of these growth factors and their receptors (ErbB/HER) is accompanied by a high expression of ADAM17 [9], [34]. The availability of growth factors in their soluble form is usually required for efficient receptor activation thus the shedding of growth factors is viewed as a major event responsible for ADAM17 pro-tumorigenic effects. We show that MC38CEA tumors derived from ADAM17-silenced cells develop slower than tumors from control cells. Surprisingly, this difference cannot be attributed to the impaired shedding of ligands for EGFR or ErbB4, whose role in tumorigenesis is well established, because MC38CEA cells appear to lack these receptors.

### Does ADAM17 contribute to MC38CEA tumor development through NRG-1 shedding?

The fact that MC38CEA cells express NRG-1 and both subunits of its heterodimeric receptor (ErbB2/ErbB3) compelled us to consider the possibility that ADAM17-dependent pro-tumorigenic phenotype of MC38CEA cells at least partially depends on NRG-1 – ErbB2/ErbB3 axis. Despite the fact that ErbB2 is ligandless and ErbB3 lacks kinase activity, the ErbB2/ErbB3 heterodimer is considered the most potent signaling receptor of the family in respect to cell proliferation and transformation [35].

Many of NRG-1 isoforms are subjected to shedding. Some studies point to ADAM17 as the major NRG-1 sheddase [26], [27], [28], [29] but other recognize ADAM9 [36], ADAM19 [37], and BACE1 [38] as the key enzymes for NRG-1 shedding. Even though MC38CEA express ADAM9 and ADAM19 as evaluated by RT-PCR (data not shown), ADAM17 must be a dominant NRG-1 sheddase in these cells as its ∼80% silencing resulted in ∼60% inhibition of NRG-1 release and decreased phosphorylation of ErbB2.

It remains unclear why NRG-1 (endogenous as well as exogenous one) did not stimulate proliferation of MC38CEA cells. The consequences of NRG-1 binding to ErbB2/ErbB3 heterodimer may vary depending on the cell type, the set and the ratio of the EGF receptor family members expressed on the cell surface, the expression pattern of intracellular signaling molecules, and probably a number of extracellular factors. It must be emphasized that NRG-1 isoforms, apart from their ability to switch on cell proliferation under certain conditions, are crucial for directing differentiation during organogenesis.

The situation in which the pro-tumorigenic potential of ADAM17-expressing cells is not correlated with increased cell proliferation, is not limited to MC38CEA colon carcinoma. We observed that silencing of ADAM17 had no effect on the proliferation rate of B16F10 melanoma cells but strongly inhibited tumor growth after s.c. injection of the cells into syngeneic mice (data not shown). Similarly, silencing of ADAM17 expression decreased the invasiveness of pancreatic ductal adenocarcinoma cells as tested *in vitro* in matrigel-coated transwell system but did not influence their proliferation [8].

### ADAM17 influences MC38CEA motility

A high migratory potential of cancer cells contributes to tumor formation and progression. Silencing of ADAM17 significantly impaired MC38CEA cell motility. Because exogenous rmNRG-1β stimulated migration of ADAM17-silenced cells, it is possible that NRG-1 shedding is to some degree responsible for MC38CEA migratory potential. However, the inability of exogenous NRG-1 to fully restore motile activity of ADAM17-silenced MC38CEA indicates that other ADAM17-dependent events may add to the migratory potential of MC38CEA cells.

We cannot reject the hypothesis that other ADAM17-dependent events may add to the migratory potential of MC38CEA cells. Cell adhesion molecules such as CD44 or L1-CAM may be involved in the migration of tumor cells and the cleavage events are required for L1- or CD44-dependent cell migration [39], [40]. Although ADAM17 is able to cleave L1 and CD44, it seems that ADAM10 and not ADAM17 is a major sheddase for these molecules and is responsible for their constitutive shedding also from tumor cells [40], [41]. ADAM10 is expressed in MC38CEA cells, which suggests that ADAM17-mediated shedding of CD44 or L1 does not contribute considerably to observed differences in cell motility. In support, we did not observe any differences in the surface expression of CD44 between the mock-transfected and ADAM17-silenced MC38CEA cells as evaluated by flow cytometry (data not shown).

ADAM17 is also a sheddase for EpCAM, homophilic cell adhesion molecule, often highly expressed on carcinoma cells [42]. It has been shown that the loss of EpCAM weakens cell-cell interactions contributing to increased migratory potential of the cells [43]. We disregarded EpCAM as a molecule decisive for the differences in MC38CEA motility observed upon ADAM17-silencing because the analysis of recorded cell movements indicated that impaired migration was characteristic even for single cells with diminished ADAM17 activity.

To sum up, at this stage of research we are unable to indicate any ADAM17-dependent events, other than shedding of NRG-1, that may affect migratory potential of MC38CEA cells.

### ADAM17 affects MC38CEA angiogenesis

We have observed enhanced angiogenesis in the mock-transfected but not in ADAM17-silenced MC38CEA. This could result directly from the significantly diminished synthesis of VEGF-A as well as MMP-9 in ADAM17-silenced cells. Both *VEGF-A* and *MMP-9* are listed among the genes whose expression may be stimulated by NRG-1 [44], [45], [46]. ADAM17 has also been shown to stimulate VEGF-A expression via the shedding of other growth factors from the EGF family [47], [48]. However, stimulation of VEGF-A expression may occur through activation of such transcription factors as AP1, NF-κB and SP1/SP3 without the involvement of growth factors' signaling [49], [50]. It is thus possible that ADAM17 substrate(s), other than NRG-1, might affect the level of VEGF-A in MC38CEA cells. However, indicating a suitable candidate among the known ADAM17 substrates remains a challenge.

### ADAM17 influences cytokine profile within MC38CEA tumor

We demonstrated that the silencing of ADAM17 in MC38CEA cells resulted in augmented accumulation of TNF, MCP-1, and IFNγ in developing tumors. Both anti-tumor as well as tumor promoting effects have been attributed to all three cytokines [51], [52], [53]. The outcome of their action may depend both on the tumor type and the state of its development, as well as on the environmental conditions, including the presence of particular cytokines, chemokines, and growth factors in the tumor milieu. Our *in vitro* experiments indicated a strong cytotoxic effect of the combination of TNF and IFNγ towards MC38CEA, which is in agreement with numerous reports showing that simultaneous action of both cytokines may lead to a strong antitumor effect [54], [55], [56].

Both TNF receptors, TNFRI and TNFRII, are ADAM17 substrates, hence one could expect that diminished activity of ADAM17 would lead to their increased surface expression, which in turn would potentiate cell sensitivity towards the ligand—TNF. Surprisingly, we observed no difference in the TNF sensitivity of mock-transfected and ADAM17-silenced cells. However, as concentrations of both TNF and IFNγ were higher in ADAM17-silenced than in mock-transfected tumors we conclude that the growth inhibition of ADAM17-silenced tumors may at least partially result from their cytotoxic/cytostatic effects. Apart from the direct cytotoxic activity of TNF and IFNγ against MC38CEA cells, the cytokines may influence *in vivo* tumor growth also through modulating immune response.

### How does ADAM17 influence anti-tumor immune response?

All IFNγ and the majority of TNF in MC38CEA tumors are produced by infiltrating immune cells, thus the differences in their concentrations between mock-transfected and ADAM17-silenced tumors must reflect differences in the subpopulations of immune cells or their activity. It remains unclear which of ADAM17 substrates expressed by MC38CEA cancer cells could trigger changes in immune response. Tumor associated antigens (TAA) could fulfill this role as ADAM17-mediated shedding of TAA might result in weaker recognition of tumor cells by the immune system. However, so far only one such antigen, namely MUC1, has been shown to be susceptible to ADAM17-mediated shedding [57] and CEA, the model TAA expressed by MC38CEA, has not been released more efficiently from mock-transfected than from ADAM17- silenced cells (data not shown).

The potential importance of other immune system-related ADAM17 substrates as IL-6R or MIC-A/B, the ligands of NKG2D receptors, in murine tumor development is questionable because of species specificity. ADAM17 is a major sheddase for human- but not for mouse IL-6R [58] and MIC-A/B are not expressed in mice at all. The identification of the ADAM17 substrate present in MC38CEA that may influence anti-tumor immune response will be a priority for the future research.

### Conclusions

Throughout the last decade ADAM17 has been recognized as a new, promising target for cancer therapy. A number of ADAM17 selective inhibitors in form of either small molecule compounds [59] or the specific anti-ADAM17 antibody fragments [60] are presently being developed. Such therapeutic approaches are directed against tumors which drive their proliferation in an autocrine manner via EGF family members. Our results indicate that ADAM17-directed therapies could be applicable towards a much wider panoply of cancers.

## Supporting Information

Text S1 **Generation of mouse recombinant His-NRG-1β1.**
(DOCX)

Figure S1 **Analysis of activity of generated rmHis-NRG-1β1.** (**A**) Analysis of ErbB2 phosphorylation. MCF7 cells were incubated for 3 h in FCS-free DMEM and then were left untreated or were stimulated for 10 min with rmHis-NRG1β1 (100 ng/ml). Protein from cell lysates (10 µg) were subjected to Western blotting and probed with anti-ErbB2-P. Data are representative of two independent experiments. (**B**) MCF-7 cells were plated in 96-well plate (5000 cells/well). Next day the medium was changed for DMEM without FCS and to some wells rmHis-NRG-1β1 was added at different concentrations. After 4 days MTT assay was performed. Data are presented as relative A545 absorbance measurements with the value obtained for control cells taken as 1. Data are from a single experiment in sextuplicates representative of two performed.
(TIF)
