# Supplementary material for: ADAM17 Silencing in Mouse Colon Carcinoma Cells: The Effect on Tumoricidal Cytokines and Angiogenesis
Source: PLoS One. 2012 Dec 10;7(12):e50791. doi: 10.1371/journal.pone.0050791 (PMC3519469; doi:10.1371/journal.pone.0050791)
Supplement: Text S1 — Generation of mouse recombinant His-NRG-1β1. (DOCX) [file pone.0050791.s001.docx]

**Generation of mouse recombinant His-NRG-1β1.**

cDNA coding for an EGF-like domain of mouse NRG-1β1 isoform was amplified from cDNA prepared from mouse brain and cloned into *Nde*I and *Eco*RI restriction sites of pET28a vector (Novagen) in frame with an N-terminal His tag. Correct insertion of cDNA was confirmed by sequencing. The protein (His-NRG-1β1) was expressed in *E. coli* strain BL21 (DE3) (Novagen) for 3 h at 37°C after induction with 1mM IPTG (Bioshop). The protein was found in insoluble form in inclusion bodies. To isolate proteins from inclusion bodies, the bacterial pellet was resuspended in 20 mM Tris-HCl pH 8.0, 5 mM EDTA, 0.5% Triton X-100, sonicated and incubated with lysozyme (1 mg/ml) for 30 min at room temperature. After addition of MgSO_4_ (final concentration 10 mM) the cell suspension was sonicated again and centrifuged (10 000g, 15 min, 4°C). The pellet was washed twice with 20 mM Tris-HCl, pH 8.0 and incubated overnight at 4°C in solubilizing buffer (6M guanidine hydrochloride, 20 mM phosphate buffer, 0.5 M NaCl, 1 mM β-mercaptoethanol, pH 7.8) and then centrifuged (10 000g, 15 min, 4°C). In order to isolate His-NRG-1β1, the supernatant was subjected to Ni-NTA affinity chromatography. The fractions containing the recombinant protein as evaluated by SDS-PAGE were pooled. Refolding was performed as follows: the protein was diluted to the final concentration of 0.2 mg/ml and dialyzed for 12 h at 4°C to 50 volumes of buffer R (20 mM phosphate buffer pH 8.0, 150 mM NaCl, 10% glycerol, 0.02% Triton X-100) containing 8M urea. Further dialysis steps were performed against buffer R enriched in 2 mM L-cysteine and containing decreasing concentrations of urea (6M, 4M, 2M, 1M, 0.5M) in subsequent steps (each step – 12 h, 4°C). Finally, the protein was dialyzed thoroughly against PBS with 10% glycerol, and centrifuged (10 000g, 15 min, 4°C). Protein concentration was estimated using Ponceau S-based densitometric assay [[1](#_ENREF_1)]. The activity of rmHis-NRG-1β1 was confirmed by analysis of ErbB2 phosphorylation and proliferation of NRG-1-sensitive MCF-7 human breast carcinoma cell line [[2](#_ENREF_2)].

REFERENCES

1. Bannur SV, Kulgod SV, Metkar SS, Mahajan SK, Sainis JK (1999) Protein determination by ponceau S using digital color image analysis of protein spots on nitrocellulose membranes. Anal Biochem 267: 382-389.

2. Pignatelli M, Cortes-Canteli M, Lai C, Santos A, Perez-Castillo A (2001) The peroxisome proliferator-activated receptor gamma is an inhibitor of ErbBs activity in human breast cancer cells. J Cell Sci 114: 4117-4126.
