# Supplementary figures and images for: ADAM17 Silencing in Mouse Colon Carcinoma Cells: The Effect on Tumoricidal Cytokines and Angiogenesis
Source: PLoS One. 2012 Dec 10;7(12):e50791. doi: 10.1371/journal.pone.0050791 (PMC3519469; doi:10.1371/journal.pone.0050791)

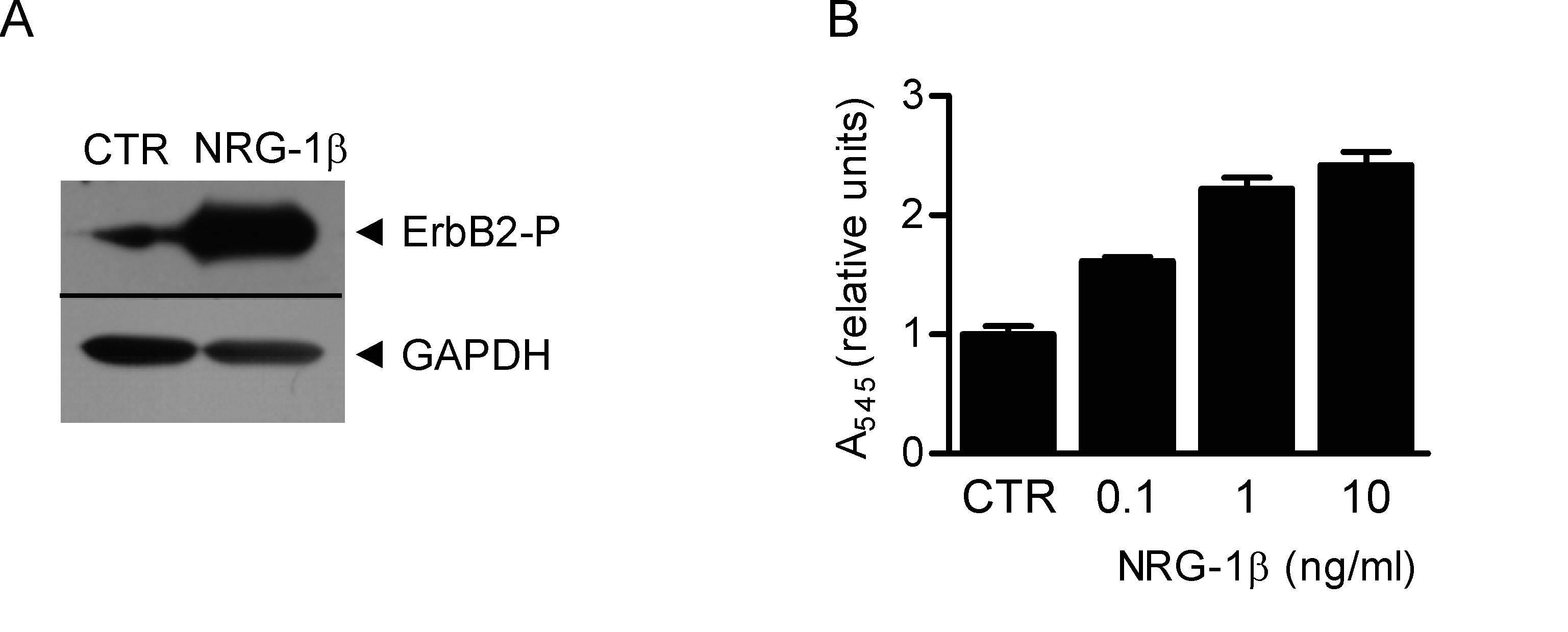

Supplement: Figure S1 — Analysis of activity of generated rmHis-NRG-1β1. (A) Analysis of ErbB2 phosphorylation. MCF7 cells were incubated for 3 h in FCS-free DMEM and then were left untreated or were stimulated for 10 min with rmHis-NRG1β1 (100 ng/ml). Protein from cell lysates (10 µg) were subjected to Western blotting and probed with anti-ErbB2-P. Data are representative of two independent experiments. (B) MCF-7 cells were plated in 96-well plate (5000 cells/well). Next day the medium was changed for DMEM without FCS and to some wells rmHis-NRG-1β1 was added at different concentrations. After 4 days MTT assay was performed. Data are presented as relative A545 absorbance measurements with the value obtained for control cells taken as 1. Data are from a single experiment in sextuplicates representative of two performed. (TIF) [file pone.0050791.s002.tif]
